# The association between birth weight and proxy-reported health-related quality of life among children aged 5 − 10 years old: A linked data analysis

**DOI:** 10.1186/s12887-021-02882-y

**Published:** 2021-09-16

**Authors:** Tahir Ahmed Hassen, Catherine Chojenta, Nicholas Egan, Deborah Loxton

**Affiliations:** 1 School of nursing and midwifery, College of Health and Medical Sciences, Haramaya University, Dire Dawa, Ethiopia; 2 Centre For Women’s Health Research, School of Medicine and Public Health, College of Health, Medicine and Wellbeing, University of Newcastle, Newcastle, New South Wales Australia; 3 Centre for Women’s Health Research, College of Health, Medicine and Wellbeing, University of Newcastle, Newcastle, Australia

**Keywords:** Birthweight, Children, Health, Quality of life, Australia

## Abstract

**Background:**

Birth weight has a substantial effect on children’s cognitive development, physical capability, and emotional development, which in turn impact on Health-Related Quality of Life (HRQoL). Generally, evidence indicates that children born with low birth weight tend to have poorer proxy-reported HRQoL, particularly at school age. However, there is limited evidence on whether variation in HRQoL exists across the entire range of possible birth weights. This study aimed to examine the association between birth weight and proxy-reported HRQoL among children aged 5–10 years old.

**Methods:**

Data from the 1973–78 cohort of the Australian Longitudinal Study on Women’s Health were linked with state-based Perinatal Data Collections and the Mothers and their Children’s Health study for 1,589 mothers and 2,092 children aged 5 − 10 years old. Generalized estimating equations were used to model the association between birth weight and proxy-reported HRQoL measured by the Pediatric Quality of Life Inventory 4.0. Results are presented as odds ratios with 95 % confidence intervals.

**Results:**

In this study, 15.61 % of children were at risk of impaired proxy-reported HRQoL. Each 100-gram increase in birth weight was associated with a 3 % reduction in the odds of impaired HRQoL (AOR = 0.97; 95 % CI: 0.94, 0.99). However, there was only limited evidence of an effect within the normal birth weight range (AOR = 0.97; 95 % CI: 0.94, 1.01).

**Conclusions:**

The findings indicate that increased birth weight was protective against impaired HRQoL, although there was limited evidence of variability within the normal birth weight range. This study contributes to the existing literature by not only emphasizing the impact of low birth weight on children’s health and health-related outcomes but also by focusing on the variability within the normal birth weight range, particularly in a setting where low birth weight is less prevalent.

## Introduction

Birth weight is an important marker of the health status of a fetus, and it has a significant impact on children’s outcomes [1]. Evidence indicates that lower birth weights (less than 2500 g), particularly extremely low birth weight, are associated with visual impairment [2], hearing loss [3], cognitive impairment [4, 5], and hyperactivity disorders [6] in young children. However, it has been noted that recognizing these impairments alone is inadequate to understand the impact and burden of low birth weights on children’s everyday life, unless a more comprehensive measure of these impairments is explored, such as Health-Related Quality of Life (HRQoL) [7].

HRQoL in children can be defined in different ways. The World Health Organisation defines HRQoL as “a child’s goals, expectations, standards, or concerns about their overall health and health-related domains” [8, 9]. Other literature defines HRQoL as a multidimensional concept that includes social, emotional, cognitive, and physical functioning as well as cultural aspects of the child and their family [10]. HRQoL can also refer to the impact of health on an individual’s overall psychological, social, and physical well-being [11]. In this paper, HRQoL has been defined as a complex multidimensional concept that includes the physical, emotional, social, and school functioning of children [12].

Several studies have explored the association between birth weight and HRQoL [7, 13]. In most of these studies, birth weight is categorized as low, normal, and high birth weight, where clinically low birth weight groups are compared to those with normal birth weight [14]. However, this approach may overlook possible associations across the full range of possible birth weights by ignoring additional variability in the outcome of interest, such as HRQoL, across the entire birth weight range [15]. Furthermore, it has also been noted that examining the effect of all possible birth weights and not just low birth weights is of particular interest because birth weight-related interventions would potentially benefit women with healthy pregnancies in addition to women with high-risk pregnancies [16].

Generally, studies have indicated that children born with a very low or low birth weight have poorer HRQoL and perform worse than their peers on specific domains of HRQoL [17, 18], although a few studies reported no differences between children with low birth weight and normal birth weight [7, 13, 14]. For instance, a systematic review found that preschool children born with very low birth weight performed worse than their peers in emotional, physical, and social functioning [19]. Another recent systematic review, however, did not find a significant difference between adults who were born with very low birth weight and those who had a normal birth weight [14]. These inconsistent findings indicate the need for a more comprehensive investigation of low birth weight and HRQoL, including accounting for variability in HRQoL that may exist within the normal birth weight range.

Various maternal and child-related factors also influence the association between birthweight HRQoL as well as early child development. For example, evidence indicated that poor maternal health and poor parenting practices negatively affected the children’s developmental outcomes around school entry which may, in turn, affect their quality of life [20, 21]. In addition, the previous studies indicated birth-related factors, for example, mode of birth affected child development, particularly cognitive performance [22]. Furthermore, child-related factors including the sex of the child and screen time also were found to affect both early child developmental outcomes and HRQoL [20, 23, 24].

Few studies have examined the variability of HRQoL [15] and other outcomes such as intelligence [16] and language development [25] across the full range of possible birth weights. The findings from these studies indicate that the variability in these outcomes exists not only within the clinically low birth weight groups but also within the normal birth weight range [25]. For example, a longitudinal study conducted in Canada that examined birth weight variability and language development showed variability in children’s language development within the normal birth weight range [25].

Despite this, the association between the entire birth weight range and HRQoL has not yet been well explored. Therefore, the objective of this study was to comprehensively investigate the association between birth weight and HRQoL by accounting for variability that may exist across the full range of possible birth weights, and also within the normal birth weight range.

## Method and materials

### Data sources

Data were sourced from the Australian Longitudinal Study of Women’s Health (ALSWH), the Mothers and their Children’s Health study (MatCH), and Australian state-based Perinatal Data Collections (PDCs).

The ALSWH is a longitudinal population-based survey that has been conducted since 1996 with three cohorts of women born in 1921-26, 1946-51, and 1973-78. Over 40,000 women responded to the baseline survey [26]. Participants were randomly selected from the Medicare database (the Australian universal health insurance system) and women from rural and remote areas were sampled at twice the rate of women in urban areas. The current study is based on the 1973-78 cohort, who have been surveyed approximately every three years.

The MatCH study is a sub study of the ALSWH. Women in the 1973-78 cohort who had reported at least one live birth were invited to complete surveys in 2016, either online or on paper, about their biological children aged under 13 years [27].

The PDCs are population-based data collections of pregnancy and birth information for each Australian state and territory. The PDC is also known as the Perinatal Statistics Collection in South Australia [28] and the Midwives Notification System in Western Australia [29]. The data include both live births and stillbirths, where gestational age is at least 20 weeks or birth weight is at least 400 g with slight variations between some states and territories [30].

### Study participants and samples

The participants of this study were 2092 children aged 5–10 years of 1589 mothers in the 1973-78 cohort of the ALSWH who participated in the MatCH study and who did not opt-out of external data linkage.

### Variables and measurements

The variables were organized using the HRQoL theoretical model that was modified for the Pediatric population [31], from the theoretical model developed by Wilson and Cleary [32]. The modified Pediatric version of HRQoL model postulated that four categories of variables, that are characteristics of individual, characteristics of the environment, symptoms status, and functional status related directly or indirectly with HRQoL [31]. Moreover, we also retained one important category, that is biological/physiological variables category, from the original theoretical model developed by Wilson and Cleary, to model the association of birthweight and proxy-reported HRQoL by accounting for various covariates listed below.

### Outcome variable

The outcome of this study was proxy-reported HRQoL, and it was measured by the Pediatric Quality of Life Inventory 4.0 (PedsQL 4.0) in the MatCH study. PedsQL is a 23-item proxy questionnaire for parents of 2–18 years old children [33]. PedsQL covers four domains: physical, emotional, social, and school functioning. Physical functioning is measured with eight items (e.g. walking, participating in sports activities, lifting heavy objects). Emotional functioning is measured with five items (e.g. feeling afraid or scared, feeling sad, and feeling angry). Social functioning is measured with five items (e.g. getting along with other children, getting teased by other children, keeping up when playing with other children). School functioning is measured with five items (e.g. paying attention, forgetting things, keeping up with school work) [34]. The PedsQL is a valid and reliable measure of HRQoL for both unwell and healthy children. Mothers were asked to rate the functioning of their children in the past month on a five-point Likert scale (0 = never; 1 = almost never; 2 = sometimes; 3 = often; 4 = almost always), and items were reversed and transformed so that the higher scores reflected better functioning. A PedsQL score is usually reported for each subdomain by taking a sum-total average of the individual item scores within the subdomain and for all subdomains together as a total PedsQL score, which is reported on a 0-100 scale, with higher scores representing better HRQoL. In this study, we considered the total PedsQL score as the primary outcome. Scores were dichotomized based on 1 standard deviation (SD) below the mean; children who fell below this cut-off point were categorized as being at risk of impaired HRQoL [34].

### Exposures

The main exposure variable in this study was birth weight. Birth weight was sourced from the PDCs. It was measured to the nearest gram and was used as a continuous predictor.

### Covariates

Based on the literature review and theoretical background, the models investigating the association between birth weight and HRQoL were adjusted for the following covariates: parenting practices (measured using the Alabama Parenting Questionnaire Short Form included in the MatCH survey), mother’s physical and mental health (measured using the SF-36 instrument), mother’s area of residence, mother’s age at birth, marital status, child’s mode of birth, child’s average screen time per day, and etc.

### Data analysis

Descriptive statistics for the maternal and child variables were reported as means and standard deviations for the continuous variables and frequencies and percentages for the categorical variables. Linearity was checked for the continuous predictors and the outcome variable, and multicollinearity was checked for the predictor variables using variance inflation factors (VIF). VIFs greater than 10 were indicative of multicollinearity. Bivariate analyses were performed to select relevant variables for inclusion in the final multivariable model. Variables that were considered clinically important or which displayed a bivariate association with HRQoL with P-values of less than or equal to 0.25 were included in the final models.

Generalized estimating equations (GEE) [35] models were fitted to examine the impact of birth weight on HRQoL, with children nested within each mother treated as having exchangeable working correlations. GEE models are a flexible regression-based approach for dealing with clustered or correlated data [35, 36]. Two models were constructed. The first model included entire birth weight and was adjusted for a wide range of maternal and child-related factors. The second model sought to determine whether there was variability in the risk of having impaired HRQoL across the range of normal birth weights. The second model was specified in the same way as the first model, except that we restricted the analysis sample to babies born with birth weights between 2500 and 4500 g.

Adjusted odds ratios with 95 % confidence intervals (CI) were presented as estimates of the effect of birth weight on impaired HRQoL after controlling for potential confounders. All analyses were performed in Stata 15.0 [37].

## Results

### Descriptive statistics of the characteristics of the sample

A total of 2,092 children aged 5–10 years were included in the primary analysis that investigated all possible birth weights. The second analysis that focused on variability within the normal birth weight range included 1981 children. The mean age of the children was 6.89 years (SD = 1.36). The overall mean birth weight for the children was 3510.19 g (SD = 515.92) and the mean birth weight for children within the normal birth weight range was 3521.54 g (SD = 412.19).

The mean HRQoL score was 87.27 (SD = 10.46) and 314 (15.61 %) children were below the 1 SD cut-off and were considered at risk of impaired HRQoL (Table 1). The mean birth weight for children who were and were not at risk of impaired HRQoL was 3460.7 (SD = 601.25) and 3515.8 g (SD = 496.51), respectively. There was little difference in the key demographics between children who were and were not at risk of impaired HRQoL. Most mothers resided in major cities (60.1 and 65.0 % for children not at risk and at risk, respectively) and were partnered (82.9 % vs. 79.8 %). Children who were at risk of impaired HRQoL were less likely to have the birth father living with the child most of the time or always (90.8 % vs. 83.5 %). Furthermore, there was a substantial difference in the proportion of children with medical problems (17.9 % vs. 47.3 %).

**Table 1:** Descriptive summary of the variables in relation to HRQoL

Factor	Impaired HRQoL	
	**Not at risk** **(n = 1698)**	**At risk** **(n = 314)**
	**N (%)**	**N (%)**
Birth weight (mean/SD)	3515.8 (496.51)	3460.7(601.25)
**Demographic factors**		
Area of residence		
Major cities of Australia	1056 (60.10)	160 (65.04)
Inner regional Australia	424 (24.13)	49 (19.92)
Outer regional / remote	277 (15.77)	37 (15.04)
Marital status		
Partnered	1465 (82.91)	197 (79.76)
Non-partnered	302 (17.09)	50 (20.24)
Birth father lives with the child		
Most of the time/always	1603 (90.82)	207 (83.47)
Sometimes	62 (3.51)	22 (8.87)
No/not applicable	100 (5.67)	19 (7.66)
**Pregnancy history**		
Mother’s age at birth		
< 35	1285 (75.68)	248 (78.98)
≥ 35	413 (24.32)	66 (21.02)
Mode of birth		
Non-Caesarean	1162 (68.43)	205 (65.29)
Caesarean	536 (31.57)	109 (34.71)
Gestational hypertension		
No	1611 (94.88)	283 (90.13)
Yes	87 (5.12)	31 (9.87)
Gestational diabetes		
No	1617 (95.23)	289 (92.33)
Yes	81 (4.77)	24 (7.67)
**Parenting factors**		
Inconsistent discipline (mean/SD)	5.76 (1.95)	6.40 (2.12)
Poor supervision (mean/SD)	3.17 (0.52)	3.27 (0.72)
Positive parenting (mean/SD)	13.46 (1.51)	13.26 (1.55)
**Maternal health**		
SF 36 Physical health (mean/SD)	91.80 (13.50)	88.65 (15.220
SF 36 Mental health (mean/SD)	77.56 (13.64)	71.09 (16.32)
**Child factors**		
Child sex		
Male	868 (51.12)	170 (54.14)
Female	830 (48.88)	144 (45.86)
Child age in years (mean/SD)	6.98 (1.37)	6.94 (1.37)
Average screen time (mean/SD)^a^	1.81 (1.07)	2.09 (1.28)
Five-minute Apgar score (mean/SD)	9.14 (0.69)	9.10 (0.62)
Gestational age (mean/SD)	39.18 (1.49)	38.97 (1.89)
Medical problems		
No	1381 (82.15)	163 (52.75)
Yes	300 (17.85)	146 (47.25)

**Notes: HRQoL**: Health-Related Quality of Life; ^**a**^Average screen time was measured in hours and reported per day

### Association of birth weight and HRQoL

In the first multivariable model, birth weight was marginally associated with HRQoL after controlling for covariates. Each 100 gram increase in birth weight reduced the odds of being at risk of impaired HRQoL by 3 % (AOR = 0.97; 95 % CI: 0.94, 0.99). In the second multivariable model which was restricted to the normal birth weight range, we observed the same effect size (AOR = 0.97, 95 % CI: 0.94, 1.01) but were not able to conclude that there was evidence of a definite association between birth weight and HRQoL due to the wider confidence interval (Table 2).

**Table 2:** Results from the final multivariable models for the association between birth weight and HRQoL among children aged 5 − 10 years

Factor	Model 1 (all birth weights)		Model 2 (normal birth weights only)	
	**Unadjusted OR (95 % CI)**	**Adjusted OR (95 % CI)**	**Unadjusted OR (95 % CI)**	**Adjusted OR (95 % CI)**
Birth weight^¥^	0.97 (0.95, 0.99)	0.97 (0.94, 0.99)*	0.98 (0.95, 1.01)	0.97 (0.94, 1.01)
**Demographic factors**				
Marital status (Ref-Partnered)				
Non-partnered	0.12 (0.93, 1.69)	1.14 (0.80, 1.63)	1.21 (0.88, 1.66)	0.88 (0.60, 1.28)
Birth father lives with the child (Ref-most of the time/always)				
Sometimes	2.40 (1.43, 4.03)	2.96 (1.63, 5.35)	2.35 (1.33, 4.0)	2.75 (1.49, 5.08)
No/not applicable	2.39 (1.53, 3.75)	2.16 (1.30, 3.57)	2.59 (1.65, 4.08)	2.39 (1.42, 4.02)
**Pregnancy history**				
Mother’s age at birth (Ref-<35 years)				
≥ 35	0.80 (0.61, 1.06)	0.80 (0.58, 1.12)	0.75 (0.56, 1.01)	0.75 (0.53, 1.06)
Gestational hypertension (Ref-no)				
Yes	1.75 (1.16, 2.64)	1.32 (0.81, 2.13)	1.68 (1.07, 2.63)	1.31 (0.78, 2.19)
Gestational diabetes (Ref-no)				
Yes	1.55 (0.98, 2.44)	1.69 (0.99, 2.87)	1.35( 0.81, 2.25)	1.59 (0.88, 2.87)
**Parenting factors**				
Inconsistent discipline^¥^	1.14 (1.07, 1.22)	1.12 (1.05, 1.21)	1.16 (1.08, 1.23)	1.14 (1.06, 1.23)
Poor supervision^¥^	1.20 (1.01, 1.42)	1.15 (0.91, 1.44)	1.2 (1.021, 1.43)	1.14 (0.90, 1.44)
Positive parenting^¥^	0.91 (0.84, 0.99)	0.89 (0.81, 0.98)	0.90 (0.83, 0.99)	0.90 (0.81, 0.99)
**Maternal health factors**				
SF 36 General health^¥^	0.98 (0.98, 0.99)	0.99 (0.98, 1.00)	0.98 (0.98, 0.99)	0.99 (0.98,1.00)
SF 36 Mental Health^¥^	0.97 (0.96, 0.98)	0.98 (0.97, 0.99)	0.97 (0.96, 0.98)	0.98 (0.97, 0.99)
**Child factors**				
Child sex (Ref-male)				
Female	0.83 (0.66, 1.04)	0.88 (0.67, 1.16)	0.83 (0.66, 1.06)	0.88 (0.67, 1.17)
Average screen time^¥^	1.22 (1.09, 1.36)	1.10 (0.97, 1.24)	1.20 (1.07, 1.34)	1.07 (0.94, 1.21)
Medical problems (Ref-no)				
Yes	3.85 (3.01, 4.93)	3.80 (2.86, 5.06)	3.71 (2.87, 4.80)	3.66 (2.72, 4.92)

**Notes**: *Significant at p-value < 0.05(birth weight); ^¥^ reported as a mean and standard deviation; **HRQoL**: Health-Related Quality of Life; **OR**: Odds Ratios; **CI**: Confidence Interval

## Discussion

This study aimed to investigate the association between birth weight and HRQoL by taking account of the variability that may exist within the normal birth weight range. The finding of our study showed that increased birth weight reduced the odds of being at risk of impaired HRQoL after adjusting for the confounding factors. These findings are corroborated with previous studies that examined the associations between birth weight and HRQoL.

However, we did not find evidence of an association between birth weight and HRQoL within the normal birth weight range. This finding is in contrast with previous studies that examined the associations between birth weight within the normal range and health-related outcomes in children. For instance, studies reported that within normal birth weight range, birth weight was associated with language abilities [38], cognitive function [39], executive functions [38], intelligence quotient at age 7 [40], academic outcomes at age 10 [41], and risk of developmental disabilities such as cerebral palsy, learning disabilities and attention-deficit-hyperactivity disorder at age 3 − 17 [42].

The discrepancy between our findings and the previous studies might be attributed to factors such as the population studied and sample size. For example, a previous study reported that the MatCH participants included in this study were more educated, healthier, and more likely to live in major cities [27] and as a result, the birth weight variability among children born to these participants might have been reduced. This was partially evidenced by the higher mean birth weight in the current study compared to that of the general population in Australia (3510 vs. 3323 g) [30]. Despite the reasonable sample size within the normal birth weight range, this may have been insufficient to detect an effect if the true effect size was smaller than anticipated by the power calculations.

The mechanisms by which birth weight is linked to different health-related outcomes including HRQoL are not well understood. However, studies have documented a linear relationship between birth weight and language [25] and executive functions [43]. These two functions may, in turn, affect other aspects of functioning. For example, while impaired language functions could affect psychosocial and academic outcomes, disruption in executive functions may lead to other adverse psychological outcomes [43] which eventually impact HRQoL.

On the other hand, the paths by which birth weight is linked to various health-related outcomes in normal birth weight children may be different to children of low birth weights [38]. For children with low birth weights, medical complications such as infection, hypoxia-ischemia, and ventricular complications are responsible for poorer health-related outcomes, mainly by reducing the size of cortical structures that are important for cognitive and behavioural functions. Evidence also indicates there is variability in the size of cortical structures among children with normal birth weight, suggesting the higher birth weight is associated with a higher cortical structure [44]. Therefore, a minor difference in birth weight, even within the normal range, may indicate a serious individual variability in neuropsychological development which, in turn, affects children’s HRQoL [38].

This study has notable strengths. The study used a nationally representative sample and adjusted for important factors that may confound the association between birth weight and HRQoL. Unlike many other studies that mainly focused on the impact of clinically low birth weight on the HRQoL, this study accounted for the variability that may exist within the normal birth weight range. Furthermore, this study also utilized a standardised and common tool to measure HRQoL in children.

This study has also some limitations that must be acknowledged. Both maternal and child data were self-reported by the mother. Importantly, as only a proxy questionnaire for parents was applied to measure HRQoL in the MatCH study, data on self-reported HRQoL were not included, warranting a further study. In addition, while the ALSWH cohorts are representative, mothers participating in the MatCH sub study had better health and health-related characteristics than the general population, which should be considered when evaluating the generalizability of these findings.

## Conclusions

These findings indicate that increased birth weight was protective against impaired HRQoL, although significant variability within the normal birth weight range was not observed. This study contributes to the existing literature by not only emphasizing the impact of low birth weight on children’s health and health-related outcomes but also by focusing on the variability within the normal birth weight range, particularly in a setting where low birth weight is less prevalent. This is important because a small shift in the distribution of birth size may impact the health and health-related outcomes in children which may also be extended to adulthood and poses additional burdens on the healthcare system.

## Data Availability

The data that support the findings of this study are available from the ALSWH but restrictions apply to the availability of these data, which were used under license for the current study, and so are not publicly available. Data are however available from the authors upon reasonable request and with permission of the ALSWH.
